# Factors that sustain indigenous youth mentoring programs: a qualitative systematic review

**DOI:** 10.1186/s12889-023-15253-2

**Published:** 2023-03-06

**Authors:** James Sanchez, Jade Maiden, Elsa Barton, Lucie Walters, Donna Quinn, Nathan Jones, Aunty Kerrie Doyle, David Lim

**Affiliations:** 1 Translational Health Research Institute, School of Health Sciences, Western Sydney University, Campbelltown, Australia; 2 College of Medicine and Public Health, Flinders University, Bedford Park, Australia; 3 Adelaide Rural Clinical School, The University of Adelaide, Mount Gambier, Australia; 4 South Western Sydney Local Health District, Liverpool, Australia; 5 First Peoples Disability Network (Australia), Sydney South, Australia; 6 University of South Australia, Adelaide, Australia

**Keywords:** Indigenous, Youth, Mental health, Mentoring, Resilience

## Abstract

**Background:**

Indigenous youth worldwide continue to experience disproportional rates of poorer mental health and well-being compared to non-Indigenous youth. Mentoring has been known to establish favorable outcomes in many areas of health but is still in its early phases of research within Indigenous contexts. This paper explores the barriers and facilitators of Indigenous youth mentoring programs to improve mental health outcomes and provides evidence for governments’ response to the United Nations Declaration on the Rights of Indigenous Peoples.

**Methods:**

A systematic search for published studies was conducted on PubMed, Embase, Scopus, CINAHL, and grey literature through Trove, OpenGrey, Indigenous HealthInfoNet, and Informit Indigenous Collection. All papers included in the search were peer-reviewed and published from 2007 to 2021. The Joanna Briggs Institute approaches to critical appraisal, data extraction, data synthesis, and confidence of findings were used.

**Results:**

A total of eight papers describing six mentoring programs were included in this review; six papers were from Canada, and two originated from Australia. Studies included mentor perspectives (n = 4) (incorporating views of parents, carers, Aboriginal assistant teachers, Indigenous program facilitators, young adult health leaders, and community Elders), mentee perspectives (n = 1), and both mentor and mentee perspectives (n = 3). Programs were conducted nationally (n = 3) or within specific local Indigenous communities (n = 3) with varying mentor styles and program focus. Five synthesized findings were identified from the data extraction process, each consisting of four categories. These synthesized findings were: establishing cultural relevancy, facilitating environments, building relationships, facilitating community engagement, and leadership responsibilities, which were discussed in the context of extant mentoring theoretical frameworks.

**Conclusion:**

Mentoring is an appropriate strategy for improving general well-being. However, more research is needed to explore program sustainability and maintaining outcomes in the long term.

**Supplementary Information:**

The online version contains supplementary material available at 10.1186/s12889-023-15253-2.

## Introduction

Article 24 of the United Nations Declaration on the Rights of Indigenous Peoples states, “Indigenous individuals have an equal right to the enjoyment of the highest attainable standard of physical and mental health”. It mandates member-states to “take the necessary steps with a view to achieving progressively the full realization of this right”. Yet, Indigenous youth worldwide continue to experience disproportional rates of poorer mental health and well-being [1–8]. Poorer mental health and well-being among Indigenous youth can be linked to general and specific stresses and challenges, structural inequalities, and many adverse determinants of health [9–11]. Many initiatives developed internationally over the past two decades have begun to recognize the importance of understanding the culture and worldviews of Indigenous peoples [12–17]. Transgenerational trauma from the historical effects of colonization, cultural oppression, and marginalization of Indigenous peoples have significantly detrimental effects on Indigenous mental health and wellbeing [18, 19]. The loss of connection to land, kinship networks, power, autonomy, memory, traditions and community, and language have facilitated a loss of belonging, a core part of the Indigenous identity [20–22]. Continued exposure to systematic racism due to colonial assimilation policies and practices fostered mistrust of mainstream health services [19, 22]. The previous deficits-based approaches to Indigenous youth mental health resilience unintentionally led to an erroneous perspective of blaming the individual [23, 24]. The current international approach to ameliorate the adverse outcomes of mental health calls for strategies that focus on integrating Indigenous worldviews and a whole of system approach [22, 25].

Mentoring has been identified as an effective strategy for establishing protective factors that promote better lifestyle behaviors and is known to enhance positive developments in social, emotional, and physical domains of health and well-being among youth in different contexts [26, 27]. Specifically, mentoring relationship between an adult and a young person contribute to the development of resilience and socio-emotional well-being. Within an Indigenous context, mentor-mentee relationships are developed through active engagements in sociocultural settings, where mentors provide psychosocial support, development, guidance, and encouragement [28, 29]. These mentor-mentee relationships seek to develop emotional connections that promote the idea of social mentoring [30]. Mentoring programs have been identified as appropriate tools for Indigenous communities that facilitate the continuation of culture and identify established partnerships with local communities [31]. By acknowledging contextual risk factors, emphasizing the potential of the individual Indigenous youth, mentoring programs can incorporate Indigenous ways of knowing, being, and learning to facilitate cultural identity development, while recognizing the community through collective ownership, responsibilities and resilience [31, 32].

There is a gap in the literature synthesizing crucial mentoring aspects that improve Indigenous youth’s mental health and well-being. A preliminary search did not identify any review publications in this context [22]. It was anticipated that a broad range of papers would be identified, encompassing different types of qualitative studies, varying geographic locations, and diverse characteristics of participants and Indigenous communities; therefore, a systematic review methodology was employed to summarize this complex evidence. This paper aims to: (i) determine the barriers and unique challenges to designing, delivering, and sustaining Indigenous youth mentoring programs for improved mental health; and, (ii) identify the facilitators and enablers of Indigenous youth mentoring program design and delivery for improved mental health.

## Methods

This review was conducted in accordance with a priori protocol [22], following the Johanna Brigg Institute (JBI) methodology for qualitative systematic review [33].

### Search strategy

Details of the search strategy and keywords have been published elsewhere [22]. Searches were performed from 2007 to July 2021 using electronic databases: PubMed (NLM), Embase (Elsevier), Scopus (Elsevier,) and CINAHL (EbscoHost). Grey literature was searched using Trove, OpenGrey, Indigenous HealthInfoNet, and Informit Indigenous Collection. Reference lists of selected papers were screened for additional studies, and the authors of included papers were contacted for further recommendations.

### Inclusion criteria

This study included papers from any geographical location, published in English, that described mentoring of Indigenous youth (defined as under 25 years old). We have respectfully referred to Indigenous people as all ethnic groups who are original inhabitants of a given region [34]. See the published protocol for further detailed definition and justification of inclusion terms of ‘Indigenous’, ‘mentor’, ‘mentee’, ‘mentoring’, and ‘youth’ [22]. Studies were included if the mentoring programs aimed to empower youth or provide support in the context of health (including mental, physical, and emotional health), holistic well-being, or support connection to community and/or family. All types of mentoring styles, including peer, group, online, and one-on-one mentoring, as well as culturally specific models (that met all other inclusion criteria) were included. All settings (e.g., community, religious, education programs) run by any organization (e.g., managed by Indigenous or non-Indigenous peoples) were included. All types of qualitative studies were included, unpublished grey literature, reports and papers in use by professional bodies, and other appropriate sources were included [22]. Quantitative studies were excluded, as it was felt that qualitative methodology would be best to capture the descriptive data of interest applicable to the research question.

Screening of eligible papers was conducted independently by two authors (JS and JM) against the inclusion criteria [22], discrepancies were discussed with two other authors (DQ, EB, LW, JS, JM, DL) until a consensus was reached. A random audit of screening was conducted by EB. A kappa coefficient, a statistical measure of inter-rater reliability, was obtained with agreement between ratings used as an indication of the reliability of the reviewer’s classifications [35]. An agreement rate of > 88% was achieved, representing a high level of agreement.

### Data extraction

Authors (JS and JM) independently extracted data in accordance with the a priori protocol [22]. A random audit of data extraction was conducted by EB and DL. Findings were discussed with other authors (DQ, NJ, EB, LW, JS, JM, DL).

### Assessment of methodological quality

Critical appraisal of included papers was conducted as per the JBI Critical Appraisal Checklist for Qualitative Research critical appraisal instrument (see Appendix 1). No papers were excluded in this review based on methodological quality. Two authors (JS and JM) completed assessments independently and audited by DL or EB. Discrepancies were discussed with other authors (DQ, EB, LW, JS, JM, DL) until a consensus was reached. There was strong agreement between reviewers (ĸ>0.8). Cultural validity of the included papers was assessed against the Aboriginal and Torres Strait Islander Quality Appraisal Tool [36].

### Data synthesis

Synthesis of data was completed using JBI meta-aggregative approach. Firstly, the extraction of findings was accompanied by illustrations from all included papers to establish a level of credibility to each finding based on the degree of support each illustration offered. These findings were then aggregated into categories according to their similarities in meaning. Categories were aggregated once again into a set of synthesized findings, which were used to generate recommendations for guidelines and practice. Cultural validation of the synthesis was conducted with a panel of First Nation researchers (JM, DQ, NJ, AKD) resulting in a strong level of agreement being reached (ĸ >0.8).

A ConQual approach was used to assess finding confidence [37], in which a score was established. Each synthesized finding is graded as high, moderate, low, or very low based on the dependability of included papers for that synthesized finding and the credibility of each finding for each category. Dependability was based on the critical appraisal scores relating to the congruency of research methodologies to the overall research process. A level of credibility was assigned to each finding as unequivocal, credible, or not supported (see Appendix 1).

## Results

**Fig. 1:**
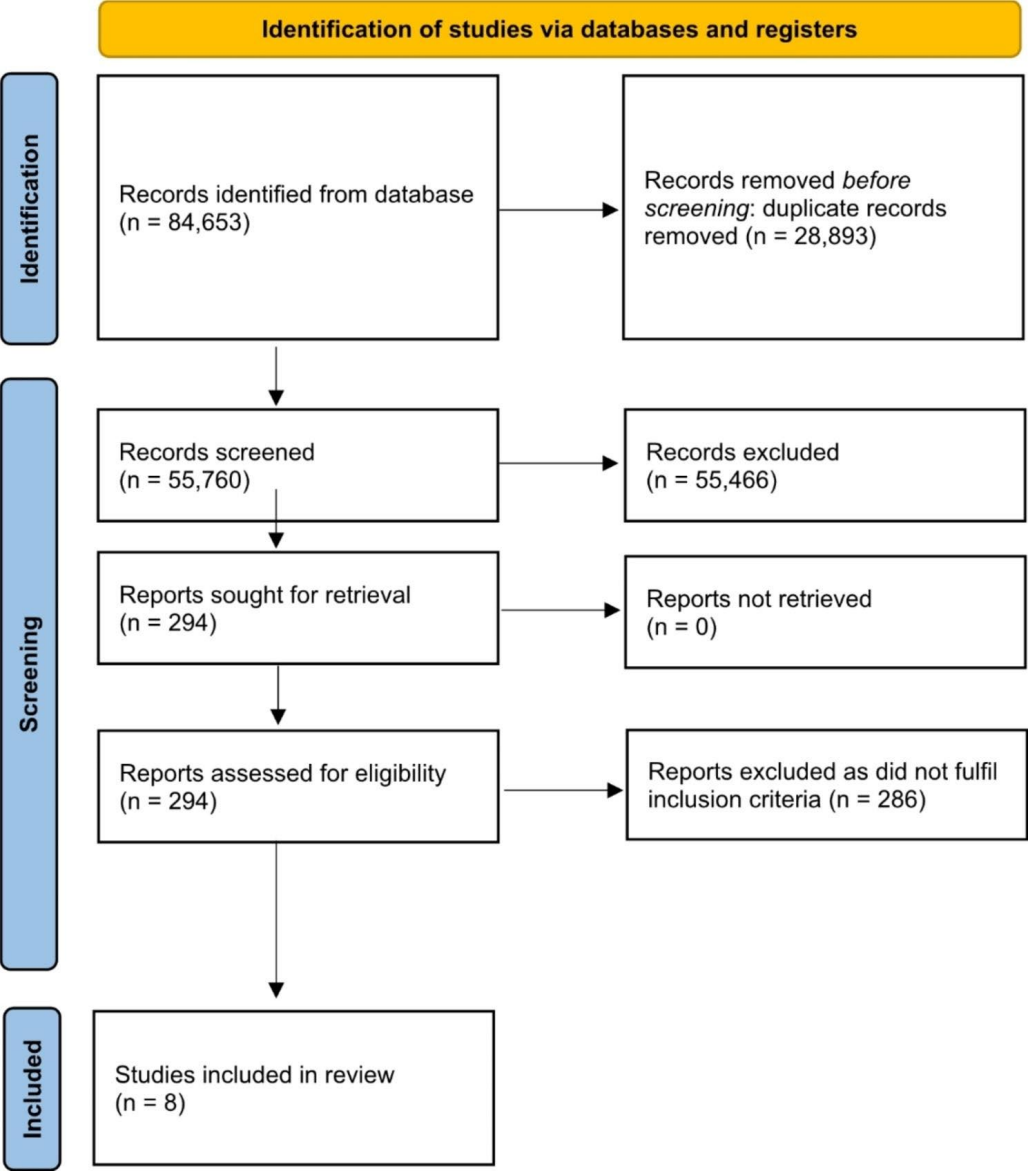
PRISMA flow diagram of literature search and selection process [59]

Figure 1 represents the PRISMA flow diagram showing the identification process of the final eight papers. A total of 84,653 hits were identified across all electronic databases and grey literature searches. After the removal of duplicates, 55,760 records were screened, 55,466 records were excluded by titles, and 286 were excluded by abstract. Six papers from Canada [38–43] and two in Australia [44, 45] were included in this review. Characteristics of included papers are summarized in Table 1. These eight papers are based on six programs: four from Canada and two from Australia. A summary of program details can be found in Table 2, including models of delivery. Four of the included papers focused on the perspectives of mentors only [38, 42–44], one study focused on only the mentees’ views [39], and three papers included both mentors’ and mentees’ perspectives [40, 41, 45]. Perspectives of various community members were included: parents, carers and Aboriginal assistant teachers [45], Indigenous program facilitators [40], Young Adult Health Leaders [42], and community Elders [43]. Three programs were implemented nationally [38, 39, 41, 42, 44], while the other programs targeted specific local Indigenous communities [40, 43, 45]. Four programs were designed around mentoring within school-based contexts [38, 39, 44, 45]. Mentoring styles involved peers [38, 39, 41, 42], groups [38, 39, 45], and one-on-one mentoring [38, 39, 44]. One program was academically focused [44], while others focused on promoting healthy lifestyles and school engagement [38, 39, 41, 42, 45].

**Table 1:** Characteristics of included papers

Paper	Program	Location	Program Target Group	Sample	Purpose/Aim	Methodology	Data Collection	Sources of rigor	Final themes
O’Shea et al. 2013. [44]	Australian Indigenous Mentoring Experience (AIME)	Australia	Aboriginal and Torres Strait Islander primary and high-school students of all ages	8 university student mentors (5 female, 3 male)	Examine the mentoring relationship between university students and Indigenous school children.	Narrative Inquiry	Digital stories - Narrative script Pre/post program Semi-structured interviews	Use of multiple data sources Member checking Cross-checking interpretations with participants	Reasons for getting involved Countering expectations, assumptions and stereotypes Making connections with Young Indigenous Mentees
Peralta et al. 2018. [45]	Government funded (Aboriginal controlled organization) sports-based program	Northern Territory, Australia	All Aboriginal students from 3 remote Aboriginal communities ranging between 80 and 240 km away from Alice Springs	13 community members (parents/carers), 21 principal/teachers, 10 Aboriginal Assistant Teachers, 27 mentors, 55 mentees	Explore how participants perceive themselves and others, and how mentoring can improve and meet the needs of remote Aboriginal community youth	Qualitative case study design	Semi-structured interviews Creative artworks	Member checking; transcripts and summary of findings	Relationships and broadening skills and exposure, increasing students’ self-esteem and aspirations, supporting school activities, school attendance and engaging with students not in school system, holistic approach, and cultural understanding
Crooks et al., 2017. [39]	The Fourth R: United Our Nations Mentorship Program	Canada	First Nations, Métis, and Inuit students grade 7–12 in southwest Ontario	28 student mentees	Longitudinal evaluation of participation on positive well-being through mental health and cultural identity	Exploratory mixed methods	Semi-structured interviews	Interviewers independent of mentoring program Assessment of inter-reliability by Cohen’s pooled κ	Intrapersonal Interpersonal Learning (Culture and healthy relationship skills)
Coyne-Foresi et al., 2019. [38]	The Fourth R: Uniting Our Nations Mentorship Program	Canada	First Nations, Métis, and Inuit students from local schools in grades 9–12	18 program mentors	Explore the benefits of mentoring from the perspective of high school youth mentors	Mixed methods approach	Semi-structured interviews	Independent reviewing of GCM cluster analysis from two researchers	Making cultural connections Benefits to self Relationships with family and friends
Fanian et al. 2015. [40]	Kts’iìhtła (“We Light the Fire”) Project	Behchokǫ̀, Northwest Territories, Canada	First Nations Tłįchǫ youth aged 15–25 in the community of Behchokǫ̀, Northwest Territories	5 Indigenous program facilitators, 4 youth program participants	Develop a community-led, youth driven model to strengthen resiliency through youth engagement through the arts	Mixed-methods approach	Observational field notes Focus groups Reflective practice	Triangulation in data analysis: independent analysis of data sources	Youth perspectives; interests in learning the arts, cultural relevancy, building new relationships Facilitator/mentor perspectives; program deliverance, access to resources
Lopresti et al., 2020. [42]	Indigenous Youth Mentorship Program (IYMP)	Canada	First Nations, Métis, and Inuit elementary school students in Canada	Interviews: 3 Young Adult Health Leaders and 1 youth mentor Focus Groups: 8 Young Adult Health Leaders and 8 youth mentors	Describe key characteristics of IYMP implementation perceived by peer youth mentors and Young Adult Health Leaders	Focused ethnography	Participatory field notes Focus groups Semi-structured interviews Meeting notes	Researcher included in IYMP national team meetings and bi-weekly teleconferences Triangulation during data analysis through multiple interpretations of the data Member checking	Building relationships Communication Open engagement Instilling sense of ownership Program supports
Ferguson et al., 2021. [41]	Indigenous Youth Mentorship Program (IYMP)	Canada	First Nations, Métis, and Inuit elementary school students in Canada	23 students, 4 mentors, 7 Young Adult Health Leaders	Explore YAHL, mentor, and elementary student experiences in the program	Qualitative Description	Sharing circles One-to-one in-depth interviews Focus groups	Peer debriefing Alternative interpretations of the data	Fostering wellness (physical, emotional, mental) Building relationships Exploring leadership
Ritchie et al. 2010. [43]	Outdoor leadership training program	Northeast Ontario, Canada	All First Nations adolescents aged 12–18 living on reserve in Wikwemikong	17 Elders, 4 mental health workers	Build Aboriginal youth resilience and cultural identity through outdoor community adventure.	Community-based participatory action research	Focus groups	Cho and Trent (2006) guidelines for transactional and transformational validity [60]	Influencing self and others Connecting with Aboriginal roots and Culture Respect and values Persistence challenges and strategies

**Table 2:** Characteristics of included programs

Program	Program Type	Mentor	Mentee	Program Aims	Delivery Method
Australian Indigenous Mentoring Experience (AIME) [44]	School-based mentoring	Volunteer university students across all disciplines	Indigenous Australian high school students	To provide Aboriginal and Torres Strait Islander high-school students educational support through safe and supportive connections with university student mentors	School-based mentoring program involving two modules; Learning Centre staff visiting schools for after-school sessions, and university visits for students to explore post-school opportunities
Government-funded program (Aboriginal controlled organization) [45]	Sports-based group-mentoring	Six male and female volunteers in each community	Aboriginal school students within three Aboriginal remote communities	Encourage school attendance and engagement, goal setting and future aspirations, sporting participation, and positive lifestyle choices among young people in communities	Sports-based mentoring program utilizing group mentoring system for remote communities for 1 week, 3 times a year
Fourth R: Uniting Our Nations Mentoring Program [38, 39]	Group mentoring School-based, one-on-one peer mentoring	First Nation young adults Student peer mentors: Grades 10–12	Indigenous students across Canada: Grades 7–9	To provide school-based, culturally relevant peer mentoring programs aimed to engage students in their schooling and encourage continued participation in formal education	16–18-week school-based peer mentorship program; First Nation young adult-led group mentoring sessions for elementary students One-to-one peer mentoring for secondary students
Kts’iìhtła (“We Light the Fire”) Project [40]	Group-mentoring	5 Indigenous facilitators (3 from the community of Behchoko)	Tłįchǫ youth aged 13–22 from the community of Behchoko, Northwest Territories	Explore the role of creative arts as a link to positive effects on health and resiliency among youth	5-day creative arts and music workshop. Program workshops were hosted by the Tłįchǫ Community Action Research Team
Indigenous Youth Mentorship Program (IYMP) [41, 42]	School-based peer mentoring	Young Adult Health Leaders (Undergraduate university students) Student peer mentors (Grade 10)	Indigenous elementary students across Canada: Grades 4–5	The IYMP integrates Indigenous values aimed at the promotion of healthy lifestyles in children and youth. The IYMP intends to build on the strengths of youth as they assume leadership roles in their community	Community driven, school-based mentoring program 1–2 days a week for 20 weeks involving physical activity/games, healthy snacks and relationship building activities
Outdoor Leadership Training Program [43]	Group mentoring	Program staff and guides from Wikwemikong	Adolescents aged between 11–19 years living on reserve in Wikwemikong	Through the *Seven Grandfather Teachings*, promote youth development by developing a sense of Indigenous identity, give opportunities for growth, and self-reliance and independence	Community-based 10-day outdoor leadership training program

### Summary of program descriptions

#### Australian Indigenous Mentoring Experience (AIME) [44]

The AIME program provided Aboriginal and Torres Strait Islander students with opportunities for educational support through connections with university volunteer-mentors in partnership with multiple institutions across Australia. Two delivery models were involved: after-school sessions in Learning Centers with local high schools that were staffed by volunteer-mentors, Indigenous students from both primary and high school received support for education and participated in relevant activities. The second model offered opportunities within local universities through on-campus interactive and leadership programs for Indigenous students in their later years of high school while offering 60 h of additional support.

#### Sports-based mentoring program [45]

Run by an Aboriginal-Controlled Community Organization, this Australian sports-based group-mentoring program was designed to engage Indigenous youth in remote communities in sports and physical activity, encouraging positive lifestyle behaviors and school engagement through active sporting activities. The program runs for one week, three times a year, within school settings by mentors consisting of athletes and people with specific capabilities. Mentors help teachers and Aboriginal Assistant teachers with classroom activities, including educating students on the importance of physical activity and health. After-school sports sessions are also run within the local communities during that time.

#### Fourth R: uniting our nations [38, 39]

This Canadian school-based peer-mentoring program was a strengths-based mentoring program that aimed to promote active engagement in schools and participation in formal education for elementary and secondary First Nations, Métis, and Inuit students to further education and pursue educational goals. Developing healthy relationships between Grades 7 to 9 students and students between Grades 10 to 12 who acted as mentors aimed to help facilitate help-seeking and self-advocacy skills, identity development through cultural activities, nurture positive relationships and enhance school engagement for all participants involved.

#### Kts’iìhtła (“We Light the Fire”) project [40]

Hosted by the Tłįchǫ Community Action Research Team as a direct response to address high rates of suicide in local Indigenous communities, the Kts’iìhtła: “We Light the Fire” project was a five-day creative art and music workshop held in the local community of Behchok (Canada) that was established to explore the role of creative arts on Indigenous youth resiliency. Built on existing capacity and established relationships with the local community, the program aimed to incorporate local values and beliefs through project collaborations and exploring critical issues within their communities experienced in their everyday lives.

#### Indigenous Youth Mentorship Program (IYMP) [41, 42]

The IYMP was a Canadian peer-led mentoring program that focused on promoting healthy lifestyles, building on the strengths of youth who assumed leadership positions in their participation throughout the program. Programs were offered to elementary students through physical activity, healthy snacks, and relationship building activities. Based on culturally relevant and theoretical frameworks, the IYMP delivery incorporated multiple stages of mentoring, where mentoring was incorporated between YAHLs, high school mentors, and elementary school mentees to facilitate learning in all directions and establish community connections.

#### Outdoor leadership training program [43]

Based on the Seven Grandfather Teachings of respect, love, wisdom, honesty, humility, and truth, the Outdoor Leadership Training Program was a ten-day community-led program designed specifically through close collaboration with local community members from the Wikwemikng community in Northeastern Ontario, Canada. Guided by local Indigenous facilitators who acted as mentors for the participants, the program involved an intensive wilderness canoe expedition - experiencing natural challenges through rapids, portages, navigation, and open-water crossings. Participants were assigned leadership responsibilities and experiences in solo components of the program.

### Synthesized findings

By exploring the perspectives of both mentors and mentees through qualitative data, five factors emerged from the data that facilitated positive improvements across multiple domains of Indigenous youth health and wellbeing. These five factors were: establishing cultural relevancy, facilitating environments, building relationships, community engagement, and leadership responsibilities. These factors were found across all programs that influenced improvements in social, behavioral, psychological, attitudinal, and academic performances of Indigenous youth, which in turn appeared to increase overall resilience in their communities.

#### Establishing cultural relevancy

Acknowledging and understanding Indigenous cultures was a key to the success of the interventions and programs focusing on the health and well-being of Indigenous youth. Whilst there was significant evidence of the positive outcomes of mentoring within the literature, there have been many instances of mistrust and doubt towards mainstream services and initiatives that were seen as individualistic and differentiate from egalitarian-based models that incorporate Indigenous worldviews.

For example, having relevant cultural frameworks embedded within the Indigenous youth mentoring program design was identified as an important factor in ensuring cultural relevancy. For instance, the Kts’iìhtła program was guided by the Elders and community representatives, integrating Tłįchǫ values and beliefs through shared community wellness goals with the Tłįchǫ Community Services Agency and Mental Health and Addictions Action Plan [40]. Likewise, the outdoor leadership training program incorporated the Seven Grandfather Teachings [43], the Fourth R program adapted the Medicine Wheel life cycles [38, 39], and the IYMP integrated Indigenous values through the Circle of Courage framework [41, 42].

Three fundamental approaches were highlighted which addressed cultural appropriateness and maintained cultural relevancy throughout the programs. Cultural competency training was important, especially for non-Indigenous mentors to understand the specific needs of local Indigenous youth, their families, and the community. Furthermore, incorporating traditional cultural teachings through partnerships with local community members allowed for the delivery of culturally safe programming and communication.

Cross-cultural training was necessary to maximize learning opportunities for volunteer-mentors in the AIME program; [44] formal training took place in the form of regular debriefing sessions between mentor and mentees to provide meaningful experiences [45]. As such, mentors, specifically those who were participating in the program for the first time, felt vast improvements in their role. Mentors of the IYMP attended national research gatherings to understand the program’s design and delivery [41, 42]. Introductions to cultural frameworks embedded within the program allowed for cultural understanding and insight into issues that mentees faced individually and in the community. This was also emphasized in the Kts’iìhtła program, that pre-program cultural training on local history and culture positively influenced cultural relevance in the delivery of the program [40].

Cultural learning and exposure to cultural teachings and practices during program delivery were important in facilitating cultural relevancy. Connections with Indigenous members of the community and the support they received were of particular importance. Participants had opportunities to learn and connect to their cultural teachings that were presented in ways that were meaningful to participants. For instance, many mentors saw the AIME as an opportunity to connect with Indigenous members of the local community, which facilitated the importance of recognizing Indigenous cultures in the delivery of mentoring programs [44].

Culturally appropriate pedagogical approaches involving community circles and storytelling that incorporated cultural and traditional values that allowed participants to reflect on their culture and how they were tied to their experiences in life. For instance, participants of the IYMP described increased awareness and knowledge of Indigenous culture by participating in traditional Indigenous games and teachings from the Traditional Knowledge Keepers [41, 42]. This promoted cultural relevancy, where participants engaged in cultural components of the program, “I felt as if I was able to connect more with what we were learning. Not necessarily that I learned more but that we were exploring it on a different level” [41 p. 741].

Cultural engagement established a sense of pride for mentors as they were able to incorporate components of their own culture for others to learn and understand. This was important as it allowed all participants to engage in aspects related to their identities.

This review suggests that youth with strong links to cultural awareness and identity resulted in an increased sense of belonging, self-confidence, autonomy, and overall functioning, suggesting protective factors against adverse mental health outcomes [39].

An important aspect of these mentoring programs was facilitating a culturally relevant strengths-based approach, with the idea of empowering both program participants and their communities through a sense of ownership and cultural identity to influence future career aspirations. As a result, programs presented mentees and youth with a strength-based mindset rather than a deficit model that encourages expectations, assumptions and stereotypes that are problematic within modern-day society. Mentors within the AIME, especially first-year mentors who had limited contact with Indigenous communities, reflected upon the differences in pre-conceived thoughts between their expectations of Indigenous youth mentees to their actual experiences within the program. One mentor described her experience as “just normal, like I could be talking to any other kid from the local feeder High School” [44 p. 402]. Many mentors mentioned learning and self-discovery, redefining their own perceptions throughout the program and were surprised at the unexpected similarities they shared with mentees. One of the Indigenous mentors’ experiences led to questioning their own appearance, exposing misgivings and insecurities about their own cultural identity: “I know I’m Indigenous, but then am I really accepted as Indigenous. I still have that battle with myself, am I accepted?” [41 p. 402].

#### Facilitating environments

Facilitating safe and secure environments for all participants was essential in designing and delivering mentoring programs. Program participants described many opportunities in developing their social networks. Participants mentioned that programs enabled them to establish new relationships and strengthen existing relationships within their local communities. These connections between mentors and mentees created a safe and secure environment for program participants, facilitating a sense of belonging within the program. One of the youth mentors in the IYMP program stated, “Once you step inside that program your entire mood will change no matter what” [42 p. 5].

Many participants mentioned the comforting environments that facilitated opportunities to build these supportive relationships, describing program settings as a safe and welcoming. One participant mentioned developing a mutual understanding between peer group members saying, “We get to talk with different people that we usually don’t talk with” [39 p. 98]. For Indigenous mentors and mentees, participation was seen as engaging with a community or family network. Participants’ involvement in the AIME, for example, presented an environment that opened opportunities for facilitating connections between mentors and mentees [44]. All participants felt they were part of a close-knit community during all stages of program delivery, describing the programs as a safe place that promoted acceptance, comfort, inclusion, and safety within the community [41]. As a result of the opportunities these programs gave in these welcoming environments, many peer relationships flourished as participants began to feel confident in their ability to communicate, shifting their approach and respect towards one another.

These relationships increased social cohesion both within and outside of the program, promoting positive changes in forming new friendships and encouraging increased participation through growth in self-confidence. The development of emotional wellness was a key outcome of program participation through building relationships with one another. These relationships provided a safe and comforting environment for program participants where engagement and trust felt natural. Participation in the program revealed opportunities to relieve stress for both mentors and participants due to the calming nature of the program. Furthermore, through the development of skills in the creative arts, youth participants in the Kts’iìhtła program found the workshop environment to provide opportunities to discuss community issues and visions for change reflected within their final art projects [40].

#### Building relationships

Mentoring programs were found to facilitate the development of relationships, including connections to people, culture, and communities. The program design was important for facilitating these connections, as mentors described naturally occurring interactions within programs. Mentoring relationships provided opportunities to discuss backgrounds, interests, personalities, and sharing of stories, all of which were important for the ongoing development of the program. When respect was reciprocal, mentees described instances of belonging to the program and the community, “The more and more I went there, the more I felt like I belonged there ‘cause it was really fun meeting all these people, playing games with them, and sharing stuff with them” [41 p.741]. However, in one instance, this was not the case, with one student mentioning that mentors “would just get mad at us and yell at us” [41 p. 741], which demonstrated that, ultimately trust is needed for mentees to engage in the program meaningfully.

Respect for mentors (compared to other authority figures) was unique, as participants mentioned the value of mentor relationships that teachers may not provide within school settings, “…Because with teachers, we do listen but with the mentors they make sure we know and we keep it locked down. Sometimes teachers don’t do that, so it doesn’t always click in. But they [mentors] always try to make sure we remember” [39 p. 98].

Involvement in these connections was not only beneficial for student participants, but was bi-directional as mentors described personal growth, self-confidence, and increased self-awareness of their community and culture as a result of participating in their mentorship role. One of the Indigenous mentors mentioned this prompted self-reflection upon what it meant to be Indigenous, as well as their own connections to family and community stating, “Looking at community initiatives that were being implemented and taking that information back to my home community” [42 p. 43].

For non-Indigenous mentors, connections with Indigenous mentees enabled a deeper understanding of educational disadvantages for non-Indigenous youth, influencing how they interacted with Indigenous communities in future engagements. Furthermore, mentors also described instances of teaching their families and those around them, mentioning the importance of teaching and providing support for those not directly involved in the program, “A lot of my family are not connected with that Aboriginal side, so it gives me the chance to explain things to them and to give them some of the knowledge that I’ve learned” [38 p. 540]. Program participants described feeling motivated into forming important relationships with their families and people in the community, “I was able to kind of find more joy in hanging out with my siblings and being with them because usually I get along with them fine but you don’t necessarily go out of your way to just interact with them” [41 p. 741].

The ability of mentoring programs to develop relationships and connections at all levels of the program also had specific influences on other positive outcomes of the program. For instance, participants of the IYMP felt that the program facilitated opportunities for learning, growth, and building a positive self-image [41].

Connecting identity through connections with others reinforced the idea of healthy relationships with others through their experiences. Developing relationships between mentees and mentors were of significant importance in providing opportunities to share and discuss culture. Student participants felt they were understood and accepted as First Nations peoples due to a shared understanding of the cultural backgrounds of the program facilitators [38]. This resulted in opportunities for honest, open dialogue where participants could open up about their culture and the challenges they face that they otherwise would not have within another setting [40].

#### Community engagement

One of the key features of program delivery was the level of engagement with the community. Strong emphasis was placed on effective communication between schools and communities for the successful implementation of the programs, including the use of appropriate language for easier participation in the program. This included simplifying language to discuss program aims that targeted community values in improving participants’ health outcomes. Participation in programs such as the IYMP was influenced by program information sheets and consent forms that were easy to understand for the students’ parents, grandparents, and guardians [41, 42].

Specific connections directly with the Indigenous facilitators and mentors were essential for program delivery due to the opportunities provided to program participants. Engagement with community members, Elders, and traditional knowledge keepers through participation in cultural activities during the programs, such as fishing and drumming, and integrating traditional Indigenous teachings through knowledge sharing, were vital for program success and ensuring cultural relevancy:

*We have often brought in like community members for the cultural activities…We made a Circle of Courage poster kind of thing where we would separate into groups and be like what does being independent mean and the kids [mentees] would come up with scenarios and then they would draw pictures and then we brought it all together as a big circle* [42 p. 6].

Programs also invited guest instructors and visitors from the community to facilitate program activities and further ties between participants and their connections to others outside the program, “I think that was really cool to bring in other people that had knowledge that we didn’t necessarily have and then we all got to do it together” [41 p. 743]. Many participants felt the need to extend these connections outside of the program by increasing relationships with those around them. In one instance, mentors of the IYMP program valued these connections and discussed ideas and ways they could give back to their own community through strategies such as volunteering [41]. Meanwhile, participants of the Kts’iìhtła program displayed interest in sharing finished projects with their community [40].

#### Leadership responsibilities

Valuing mentor roles was seen as a factor for driving successful program implementation. Some peer mentors described the challenge in developing leadership qualities as having to step outside of their comfort zones in their ability to learn, grow, and embrace their roles in leading group activities [41]. Program mentors generally discussed their own development through their roles as mentors, stating, “I make better choices because it reflects the type of mentor I am” and “becoming a mentor helps me to make good life choices and wiser choices” [38 p. 540]. Mentors felt increased responsibilities in their leadership positions, mentioning beliefs in their roles as leaders within the community, their interests in helping other people, and their overall self-perception. An increase in self-confidence and self-esteem inspired mentors to further strengthen their connections with their peers and to continue facilitating positive relationships during the program: “I think that was my favorite thing, is like seeing the way they look up at us and like how much they actually value like, what we’re teaching them” [41 p. 743].

An important aspect of the programs was the skills and knowledge that mentors brought into the community. Mentors were seen as role models for the program mentees, exposing them to opportunities available outside their local communities. Mentees were given a chance to explore different skills and experiences within mentorship programs, with the aim of giving youth a sense of empowerment through participation in program activities. Mentees were encouraged to engage with successful mentors and role models, “The more they’re exposed to the reality of what is possible for them, and the more they see people from their own culture being successful in that area who still had their culture … people who still are able to connect with them, that’s very empowering” [45 p. 39].

## Discussion

This review examined current literature on Indigenous youth mentoring programs and found five facilitating factors in the design, delivery, and sustainability of these programs to improve general mental health and wellbeing. These factors, found across all programs, demonstrated positive influences in social, behavioral, psychological, attitudinal, and academic performances of Indigenous youth, which in turn suggested increased overall resilience in these communities. Theoretical implications of the findings are discussed below, with reference to extant literature within the context of cultural relevancy, facilitating environments, building relationships, community engagement and leadership aspects of mentoring.

The significance of ensuring cultural relevancy within the programs can be considered in reference to the Multicultural Feminist Mentoring theory, which suggests that in order for mentors to have multicultural competence in meeting the unique needs of diverse mentees, the skewed power structure of the dyadic relationship should be recognized and the mentee should be equipped with tools to be empowered when encountering systemic racism [46]. Mentors in the participating programs suggested reflection on this concept:

*I don’t want to be an ignorant person… I just wanted to personally come into closer contact with it so I understood for myself what it meant, rather than just – a lot of the ways that Indigenous people are treated in Australia it’s very distant. I just don’t think that’s helpful and I just don’t want to be ignorant about it* [44 p. 400].

It is important to consider the mentee, the mentor and the mentoring relationship being affected by a series of social and cultural contexts, which can either empower or disenfranchise the mentee [47]. This perspective on mentoring advocates for an explicit concern for one’s holistic growth and well-being at the intersections of work and family lives [48], which links into community supports contributing to the success of the discussed programs.

Within the concept of facilitating safe environments, the Holding Environment theory describes how mentoring relationships can be viewed as a type of anchoring relationship. The application of this theory “yields the prediction that the presence of a high-quality relationship should moderate the expected negative association between ambient racial discrimination and organizational commitment” [49 p. 215]. It is evident that the described mentoring programs did provide a holding environment (as described by Ragins et al., [49]) for Indigenous youth by offering mentees a contained and safe space to share their experiences and reactions, providing empathic acknowledgment by accepting and validating mentee’s feelings, and offering enabling perspectives that helped mentees make sense of the situation within a non-judgmental and validating context.

Building mentoring relationships where mentors are seen as trustworthy and reliable sources of support allows for positive youth development and overall resilience [50, 51]. High-quality mentoring can be defined as being characterized by trust, disclosure, vulnerability and commitment which offers significant opportunities for personal learning, growth and discovery for all parties [52]. Mutual learning can be interpreted in reference to Relational Mentoring theory, which expands the scope of mentoring to a bi-directional, mutual, interdependent, generative, and developmental relationships [53]. This results in mutual authenticity, empathy and empowerment between mentor and mentees [53]. This review found that mentors who were more trusting and respectful were more flexible in meeting mentees’ needs and showed greater commitment to the community. Many participants in this review found a greater sense of empowerment after participating in mentoring programs that provided opportunities to develop important life skills and competencies, mainly through areas of school engagement, education, and general health and wellbeing. In addition, many Indigenous youth who developed feelings of perceived social support within mentoring programs demonstrated help-seeking behaviors beyond the program through family and other members of the community. This can then flow on to increased overall resilience in these communities.

Resilience is often tied to strong family and community support networks that establish a sense of belonging and identity, and active engagement in community activities promote positive lifestyle outcomes [54]. Resilience theory suggests the ability to avoid certain behaviors, lifestyle choices, and attitudes that threaten physical, mental, and spiritual health at times of adversity is reflective of an individual’s access to protective resources [29]. This necessitates the importance of understanding the ways Indigenous youth interact with their surrounding environments, and developing integrated models of mentoring with family and community links. Mental health is not only an outcome for individual youths but as a collective, also a determinant of community health within an Indigenous context [55]. Studies have found an overlapping connection involving individual, family, and community aligns with holistic views of Indigenous cultures, where both individual and community factors are reflective upon one another [56]. One of the main findings of this review was the importance of partnerships with established members of the community. Integrating community members such as the Elders and Traditional Knowledge Keepers in mentoring programs facilitated relationships which aid in restoring connections to ancestral communities that are threatened by a lack of connectedness [57]. Developing resilience within communities also includes incorporating factors of community engagement that emphasize the overall importance of mentoring programs that are community-driven, provide community ownership and buy-in, and promote empowerment towards positive community health and wellbeing.

Finally, this review found that the leadership aspect of mentoring by facilitating engagement and leading by example provided positive impacts on mentees and maintained healthy ongoing relationships. It has been shown that mentoring others can improve individuals’ leader identity and leader self-efficacy [58]. This research found that in turn, this influenced mentees in developing help-seeking behaviors due to their belief in their mentor peers. The sense of ownership experienced by participants ensured sustaining program success through increased participation and retention of the program in the long term.

### Recommendation for future practice and research

1. Program designers should look to empower Indigenous communities through strong community partnerships, collaboration in design and implementation, and promoting Indigenous-led, culturally appropriate programming that facilitates community buy-in and community resilience.
2. Learning from and effective integrating international models of mentoring programs may influence the success of Indigenous youth mentoring programs. There is a need to assess homogeneity and practicability in the local context.
3. This paper discussed positive outcomes of mentoring programs on general youth wellbeing however more research is needed to further quantify the impact that may have on mental health. Measuring the associated risk and protective factors of resilience through both quantitative and qualitative outcomes can provide meaningful analysis within this field.

### Limitations

There was potential for publication bias in limiting recruitment of potential studies that did not meet the strict inclusion and exclusion criteria. As this review focused on qualitative papers only, the findings were limited to factors relating to mentoring program experiences, excluding quantitative findings that may provide objective indicators of improvements in mental health and overall resilience through validated tools. Furthermore, our review only included results from studies that originated in Australia and Canada. This was surprising as many papers have explored mentoring as an approach to addressing disproportionate rates of suicide and overall health in countries such as America, New Zealand, and Greenland. As this review aimed to include studies based in these regions, findings may not be relevant due to potential differences in local community needs relating to specific cultural values and traditions, structural and language/ heritage/ cultural barriers, nuanced experience with colonization, displacement, dispossession, economic exploitation, racism, and marginalization, local priorities and preferences, and local solutions that have been trialed.

Furthermore, analysis in cross-cultural or cross-national differences between the programs, as well as any potential effect of Indigenous compared to non-Indigenous mentors were not conducted as a convergent model was used to synthesize similarities between programs. These reduce the sensitivity of the analysis in picking up unique nuances such as Indigenous versus non-Indigenous mentors and reduce the generalizability in applying findings to other Indigenous cultures; however, it is hoped that analyzing commonalities will increase the specificity of the findings which may apply to other First Nations countries not explicitly stated in the review since despite culture differences, Indigenous people around the world share common problems and experienced disadvantaged and vulnerability. Another limitation that was found to be problematic across most studies was issues with small sample sizes. Small samples of Indigenous youth participation in mentoring programs specifically make it difficult to generalize findings from the sample population to the community.

## Conclusion

This qualitative systematic review explores the perspectives of program facilitators, mentors, and mentees on factors that sustain Indigenous youth mentoring programs in varying contexts. Mentoring has shown to be a successful strategy that can be implemented within Indigenous contexts when culturally relevant approaches are appropriately implemented. Whilst mentoring in isolation has proven to result in positive outcomes, more research is needed on mentoring as part of broader initiatives in addressing Indigenous health and wellbeing.

Mentoring programs that are culturally tailored to meet the needs of the local community is the best approach to successful mentoring regardless of program aims and objectives. Holistic approaches involving strong community partnership links that build upon the strengths of Indigenous youth have provided the best outcomes on mental health and overall resilience of Indigenous youth and their communities regardless of their surrounding environments. Whilst mentoring programs have resulted in positive social and emotional wellbeing in the short term, more research and organizational commitment is needed to sustain these programs for long-term benefits.

### Electronic supplementary material

Below is the link to the electronic supplementary material.

Supplementary Material 1

## Data Availability

The datasets used an analyzed during the current study are available from the corresponding author on reasonable request. All data generated and analyzed during this study are included in this published article.

## Appendix 1. Critical appraisal and level of credibility analysis

**Table:**

	JBI Critical Appraisal Checklist for Qualitative Research	O’Shea et al. [40]	Peralta et al. [41]	Coyne-Foresi et al. [42]	Crooks et al. [43]	Fanian et al. [44]	Ferguson et al. [45]	Lopresti et al. [46]	Ritchie et al. [47]
1	Congruity between the stated philosophical perspective and the research methodology	N	N	N	N	N	Y	N	Y
2	Congruity between the research methodology and the research question or objectives	Y	Y	Y	Y	Y	Y	Y	Y
3	Congruity between the research methodology and the methods used to collect the data	Y	Y	Y	Y	Y	Y	Y	Y
4	Congruity between the research methodology and the representation and analysis of the data	Y	Y	Y	Y	Y	Y	Y	Y
5	Congruity between the research methodology and the interpretation of the results	Y	Y	Y	Y	Y	Y	Y	Y
6	Statement locating the researcher culturally or theoretically	N	Y	N	N	Y	N	N	N
7	Is the influence of the researcher on the research, and vice-versa, addressed?	N	Y	N	N	Y	Y	Y	N
8	Are participants, and their voices, adequately represented?	Y	Y	Y	Y	N	Y	Y	N
9	Is the research ethical according to current criteria or, for recent studies, and is there evidence of ethical approval by an appropriate body?	N	Y	Y	Y	N	Y	Y	N
10	Do the conclusions drawn in the research report flow from the analysis, or interpretation, of the data?	Y	Y	Y	Y	Y	Y	Y	Y

**Table:**

Included papers	Dependability grading (based on above Q2-4, 6–7)	Credibility finding	ConQual Score
O’Shea et al. [40]	Moderate (3 yes responses)	Mixed (Credible)	Low
Peralta et al. [41]	High (5 yes responses)	Mixed (Credible)	Moderate
Coyne-Foresi et al. [42]	Moderate (3 yes responses)	Mixed (Credible)	Low
Crooks et al. [43]	Moderate (3 yes responses)	Mixed (Credible)	Low
Fanian et al. [44]	High (5 yes responses)	Mixed (Credible)	Moderate
Ferguson et al. [45]	High (4 yes responses)	Mixed (Credible)	Moderate
Lopresti et al. [46]	High (4 yes responses)	Mixed (Credible)	Moderate
Ritchie et al. [47]	Moderate (3 yes responses)	Mixed (Credible)	Low
