# Supplementary material for: Factors that sustain indigenous youth mentoring programs: a qualitative systematic review
Source: BMC Public Health. 2023 Mar 6;23:429. doi: 10.1186/s12889-023-15253-2 (PMC9987150; doi:10.1186/s12889-023-15253-2)
Supplement: Supplementary file 1 — Supplementary Material 1 [file 12889_2023_15253_MOESM1_ESM.docx]

Appendix 1. Critical appraisal and level of credibility analysis

|  | **JBI Critical Appraisal Checklist for Qualitative Research** | **O’Shea et al [40]** | **Peralta et al [41]** | **Coyne-Foresi et al [42]** | **Crooks et al [43]** | **Fanian et al [44]** | **Ferguson et al [45]** | **Lopresti et al [46]** | **Ritchie et al [47]** |
| --- | --- | --- | --- | --- | --- | --- | --- | --- | --- |
| 1 | Congruity between the stated philosophical perspective and the research methodology | N | N | N | N | N | Y | N | Y |
| 2 | Congruity between the research methodology and the research question or objectives | Y | Y | Y | Y | Y | Y | Y | Y |
| 3 | Congruity between the research methodology and the methods used to collect the data | Y | Y | Y | Y | Y | Y | Y | Y |
| 4 | Congruity between the research methodology and the representation and analysis of the data | Y | Y | Y | Y | Y | Y | Y | Y |
| 5 | Congruity between the research methodology and the interpretation of the results | Y | Y | Y | Y | Y | Y | Y | Y |
| 6 | Statement locating the researcher culturally or theoretically | N | Y | N | N | Y | N | N | N |
| 7 | Is the influence of the researcher on the research, and vice-versa, addressed? | N | Y | N | N | Y | Y | Y | N |
| 8 | Are participants, and their voices, adequately represented? | Y | Y | Y | Y | N | Y | Y | N |
| 9 | Is the research ethical according to current criteria or, for recent studies, and is there evidence of ethical approval by an appropriate body? | N | Y | Y | Y | N | Y | Y | N |
| 10 | Do the conclusions drawn in the research report flow from the analysis, or interpretation, of the data? | Y | Y | Y | Y | Y | Y | Y | Y |

| **Included papers** | **Dependability grading  (based on above Q2-4, 6-7)** | **Credibility finding** | **ConQual Score** |
| --- | --- | --- | --- |
| O’Shea et al [40] | Moderate (3 yes responses) | Mixed (Credible) | Low |
| Peralta et al [41] | High (5 yes responses) | Mixed (Credible) | Moderate |
| Coyne-Foresi et al [42] | Moderate (3 yes responses) | Mixed (Credible) | Low |
| Crooks et al [43] | Moderate (3 yes responses) | Mixed (Credible) | Low |
| Fanian et al [44] | High (5 yes responses) | Mixed (Credible) | Moderate |
| Ferguson et al [45] | High (4 yes responses) | Mixed (Credible) | Moderate |
| Lopresti et al [46] | High (4 yes responses) | Mixed (Credible) | Moderate |
| Ritchie et al [47] | Moderate (3 yes responses) | Mixed (Credible) | Low |
